# Enhancement Effect of Microbubble-Enhanced Ultrasound in Microwave Ablation in Rabbit VX2 Liver Tumors

**DOI:** 10.1155/2020/3050148

**Published:** 2020-01-23

**Authors:** Shuyi Xiao, Zhiwen Hu, Yan He, Hai Jin, Yuwen Yang, Liping Chen, Qiaoli Chen, Qiong Luo, Jianhua Liu

**Affiliations:** ^1^The First Affiliated Hospital, Jinan University, Guangzhou, China; ^2^Department of Medical Ultrasound, Guangzhou First People's Hospital, Guangzhou Medical University, Guangzhou, China; ^3^The Second Affiliated Hospital of South China University of Technology, Guangzhou, China

## Abstract

**Objectives:**

One reason for the high recurrence and metastatic rates of tumors such as hepatocellular carcinoma (HCC) treated by microwave ablation (MWA) is the presence of residual foci in the tumor due to heat sink effect. Microbubble-enhanced ultrasound (MEUS) can noninvasively disrupt and block the tumor blood perfusion and has the potential to overcome the heat sink effect and enhance the therapeutic effect of MWA. The study aimed at evaluating the potential additional benefit of microbubble-enhanced ultrasound (MEUS) in hepatocellular carcinoma (HCC) treated by microwave ablation (MWA).

**Methods:**

In this study, a new strategy of combining MWA with MEUS for treating HCC was proposed. Twenty-four rabbits with VX2 tumors in livers were randomly divided into MEUS + MWA, MEUS alone, MWA alone, and blank control groups, respectively (*n* = 6). In the MEUS group, the tumors were directly exposed to therapeutic ultrasound for 5 min with a concurrent intravenous injection of microbubbles (0.1 ml/kg diluted into 5 ml saline). In the MWA group, the tumors were treated by MWA for 1 min. In the MEUS + MWA group, tumors were ablated by MWA for 1 min after ultrasound cavitation enhanced by microbubbles as in the MEUS group. In the blank control group, the tumors received probe sham and intravenous saline. Contrast-enhanced ultrasound (CEUS) was performed before treatment and immediately after treatment to display the size, shape, and contour of the tumors. Throughout the treatment process, the local temperature of the treatment area was detected by a temperature needle punctured into the tumor. The blood samples of animals were obtained after treatment for evaluating the liver function. Tumor cell necrosis and apoptotic rates were observed after treatment by histological examination.

**Results:**

CEUS showed that although perfusion defects appeared in all the treatment groups, especially in the MEUS + MWA group, there was no significant difference between the two groups on the volumes of perfusion defects, which were 1.78 ± 0.31 (cm^3^) in the MWA group and 1.84 ± 0.20 (cm^3^) in the combined group (*P* < 0.01). The time to reach the peak temperature of the treatment area was 21.7 ± 5.0 (s) in the MWA group and 10.3 ± 5.0 (s) in the MEUS + MWA group (*P* < 0.01). The time to reach the peak temperature of the treatment area was 21.7 ± 5.0 (s) in the MWA group and 10.3 ± 5.0 (s) in the MEUS + MWA group (*P* < 0.01). The time to reach the peak temperature of the treatment area was 21.7 ± 5.0 (s) in the MWA group and 10.3 ± 5.0 (s) in the MEUS + MWA group (

**Conclusions:**

These results suggested MEUS treatment alone may significantly reduce tumor blood perfusion and led to a sharp rise in the local temperature of the treatment area to a higher PT using MEUS + MWA with higher rates of necrosis and apoptosis of cancer cells without severe liver function damage, which might be a safe strategy for treating HCC.

## 1. Introduction

Hepatocellular carcinoma (HCC) is one of the leading causes of cancer-related deaths [1]. Current treatment strategies for HCC include surgical treatment, thermal ablation, and localized embolization chemotherapy alone or in combination [2]. Among these, surgical treatment is the most important and effective treatment for HCC at present, which includes surgical resection and liver transplantation [3]. The 5-year survival rate of patients undergoing surgical resection is as high as 70%, while the treatment is limited to HCC patients without hepatocirrhosis, which comprises about 20–30% of patients with HCC [4]. Despite a 4-year overall survival rate of 85% and a recurrence-free survival rate of 92%, liver transplantation is still limited due to strict criteria, surgical candidacy, tumor burden, and the availability of donors [5].

Thermal ablations such as microwave ablation (MWA), radiofrequency ablation (RFA), and high-intensity focused ultrasound are important complements of surgical treatment for HCC. Thermal ablation kills the tumors by increasing the temperature of solid tumors through heat accumulation [6]. This method has obvious advantages with regard to safety (less invasive), good tolerance, repeatability, and efficiency. HCC nodules are considered as the most common targets of thermal ablation clinically [7, 8]. Microwave ablation MWA causes irreversible thermal necrosis of the tissue through the delivery of microwave energy. Previous studies have reported that MWA can treat HCC nodules which are larger than 3 cm, resulting in a complete ablation rate of 92.6%, local recurrence rate of 22%, and 3-year survival rate of 30.9% [9, 10]. According to previous studies, “heat sedimentation effect” is one of the major factors that influence the ablation size and shape, leading to the local residual focus of the tumors. Blood flow through tumors or major peripheral blood vessels promoted heat loss and prevented heat deposition by removing the heat [11], causing a slow or insufficient temperature rise in the treatment area. Due to this, the tumor cells cannot be completely ablated after treatment and the residual foci may lead to recurrence. How to acquire a sufficient ablation area for HCC treatment has become a major issue in the use of MWA technique [12].

One of the strategies to achieve a more thorough thermal ablation area is to block the blood flow of tissues before ablation. If the blood supply of HCC and surrounding liver tissues is reduced and the “heat sedimentation effect” is reduced, the efficiency of heat ablation will be improved [13, 14]. Transarterial embolization or chemoembolization (TAE/TACE) can reduce blood perfusion by slowing down blood flow, causing local ischemia and increasing heat retention [15, 16]. This has been performed in combination with RFA and MWA, resulting in an improved complete ablation response and long-term survival rate [17, 18]. Several studies have reported the use of microbubble-enhanced ultrasound (MEUS) in the disruption of tumor microvasculature [19–21]. The inertial cavitation induced by high-amplitude, low-intensity ultrasound and microbubbles severely damages the small vessels and vasculature, resulting in the cessation of circulation in relevant tissues [22]. According to a previous study, MEUS was applied to disrupt tumor microvasculature and arrested tumor perfusion for up to 24 h [20]. The combination of MEUS and percutaneous ethanol ablation (PEA) increased the necrosis rate of tumor in rats significantly from 81.0% to 97.5% [23]. In normal rabbit liver, MEUS blocked the circulation for 15–60 min and enlarged the PEA ablation volume up to 10-fold [24]. So, MEUS combined with PEA can obviously enlarge the ablation area [25].

Hence, this study aimed at investigating the possibility, safety, and effectiveness of MEUS-induced perfusion blockage to enhance MWA of HCC in vivo.

## 2. Materials and Methods

### 2.1. Experimental Design

Twenty-four New Zealand rabbits weighing 2.6 kg to 3.0 kg, regardless of gender, were included in this study. They were purchased from the Medical Animal Experimental Center of Guangdong (MAECG) and were fed with standard laboratory diet and tap water ad libitum. The experiments on laboratory animals were performed with the approval of the Institutional Animal Care and Use Committee (IACUC) of the MAECG, Guangdong, China. Fourteen days after tumor implantation, 24 rabbits with palpable tumors (approximately 1.5–2.0 cm) were randomly divided into four groups, with 6 rabbits in each group: (i) blank control group; (ii) MEUS treatment group; (iii) MWA treatment group; and (iv) MEUS + MWA treatment group. The animals were anesthetized by using 0.3 mL/kg of 2% pentobarbital before surgery and then were placed in supine position for removing the upper abdominal hair. After a middle surgical incision of the abdominal wall, the liver lobes with tumor nodules were exposed and fixed ex vivo in situ. In the MEUS group, the liver tumors were treated with ultrasound cavitation therapy for 5 minutes combined with an intravenous injection of diluted SonoVue saline microbubble suspension at a dose of 0.1 mL/kg. In the MWA group, the liver tumors were treated with MWA for 1 min. In the MEUS + MWA group, the liver tumors were treated by ultrasound cavitation therapy firstly followed by MWA on the same target region. In the blank control group, rabbits were injected with the same amount of saline and received no ultrasound or MWA treatment. CEUS was performed before treatment and immediately after treatment to display the size, shape, and contour of the tumors and the size, shape, and contour of the effective treatment areas. Throughout the treatment process, the local temperature of the treatment area was detected by a temperature needle punctured into the tumor. The blood samples of animals were obtained before and immediately after the treatment to evaluate liver function. Tumor cell necrosis and apoptotic rate were observed after treatments by histological examination.

### 2.2. Rabbit VX2 Liver Tumor Model

**Figure 1:**
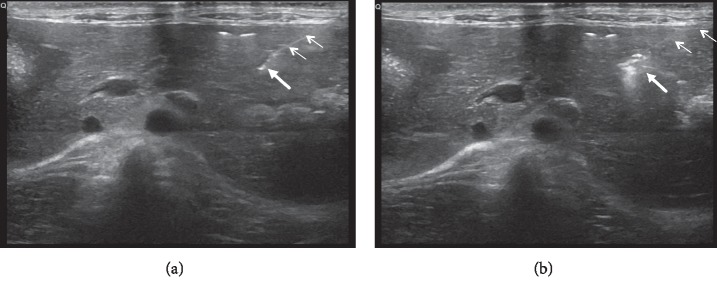
Ultrasound image of the established model by interventional method. Ultrasound-guided percutaneous puncture of 18G PTC needle into the liver, and the small arrows mark the needle path and the thick arrow marks the tumor tissue of the target area (a). PTC needle tip of the high echo mass is pushed into the liver tumor tissue fragments and is accompanied by a small amount of gas, where the small arrows mark the needle path and the thick arrow marks the tumor tissue (b).

The rabbit VX2 tumor liver model was established by an interventional method as described previously [26]. Firstly, we removed the VX2 tumor tissue from the thigh of a tumor-bearing rabbit and cut into 3-4 mm^3^ cubes under sterile conditions. Then the tumor was placed in normal saline and sliced into 1 to 2 mm^3^ fragments. Next, the 26 animal recipients were anesthetized with an intramuscular injection of 2% pentobarbital at a dose of 0.2 mL/kg, and their upper abdomens were cleanly shaved for undergoing a preliminary ultrasound (Mindray M7, Myry Biomedical Electronics Co., Ltd., Shenzhen, China) examination using a linear array transducer to determine the target implantation site within the liver. We used an 18G PTC puncture needle (Hakko, 1490 O-aza, Japan) to percutaneously puncture the liver tissue (Figure 1(a)), which included a hollow core and a sharp, blunt inner stylet. The sharp inner stylet was removed, and a small tumor fragment (1-2 mm^3^) was pushed through the core into the liver. A hyperechogenic area representing the tumor fragment was visible with ultrasound imaging (Figure 1(b)). After implantation, CEUS was performed to monitor the growth of the tumor and to measure the tumor size.

### 2.3. Ultrasound Cavitation Therapy

We used an ultrasound cavitation device with an ultrasound therapy apparatus (Full Digital Ultrasound Microbubble Cavitation Therapy Instrument, Shenzhen WELL. D Medical Electronics Co., Ltd., Shenzhen, China). A probe with a 1 MHz central frequency was used to transmit 400-cycle pulses at a 10 Hz pulse repetition frequency for 5 min. The transducer works in an intermittent mode of 5 s on and 5 s off. The actual working duty cycle was approximately 0.20%. The transducer generated a peak positive pressure of 1 MPa. All rabbits were anesthetized with an intravenous 2% pentobarbital dose of 0.2 mL/kg injected into the ear vein with a 21-gauge needle. The cavitation probe was then placed on the exposed surface of the liver tumor. In the MEUS group, the cavitation therapy was combined with a continuous intravenous injection of SonoVue saline solution (0.1 mL/kg) at the same time through the ear vein.

### 2.4. MWA Ablation

MWA was performed by using the microwave therapeutic instrument (EC0-100C, Microwave Therapeutic Instrument, Yigao Medical Equipment Co., Ltd., Nanjing, China) that is equipped with an ablation needle (ECO-100AI3, Microwave Therapeutic Instrument, Yigao Medical Equipment Co., Ltd., Nanjing, China) whose diameter is 1.6 mm. The treatment needle was inserted into the centre of the tumor nodule and its tip is ready to puncture into the deeper margins of the tumor. MWA was performed with an output power of 20 W for 5 s, and then, the needle was withdrawn.

### 2.5. CEUS and Size Measurements

CEUS imaging of the rabbit liver was performed before and after therapy. The SonoVue microbubble suspension (0.05 mL/kg) was dissolved in 5 mL saline and injected as a bolus dose into the ear vein, followed by 2 mL saline. A commercially available ultrasound imaging system (GE Vivid E9, GE Medical Health Co. Ltd, USA) that was equipped with an L9 high-frequency linear array probe was used for CEUS. Contrast modality was conducted by using a low mechanical index (MI = 0.05), and the frequency was 5–9 MHz. The ultrasound probe was placed on the surface of the liver. The CEUS images were digitally stored for up to 5 min to perform a quantitative analysis off-line. Grayscale ultrasound was used to determine the location, echogenicity, and volume of the tumor. CEUS images were used to determine the location, echogenicity, and volume of the effective treatment area. The maximum length, width, and thickness of the tumors and the unenhanced perfusion defects of CEUS images were determined from the largest slices. The volume was calculated by using the following formula: volume = *π* (length × width × thickness)/6 [27].

### 2.6. Temperature Monitoring

During the treatment, one needle thermometer (SENDAE-115, Guangzhou Sun-Gun Corp., Guangzhou, China) was inserted into the liver tumors, just near to the tip of the ablation needle or in the centre of ultrasound irradiation area. The thermometer automatically recorded the local temperature changes during the treatment.

### 2.7. Blood Sample Collection

Blood samples of animals in each group were obtained through the auricular artery or the external jugular vein immediately after treatment. The main liver function indicators such as alanine aminotransferase (ALT), aspartate aminotransferase (AST), R-glutamyl transferase (r-GT), and total protein (TP) were measured.

### 2.8. Histological Examination

The rabbits were sacrificed immediately after treatment. Liver lobes with tumor nodules were harvested, fixed in formalin, and then embedded in paraffin. Samples were cut into 4 mm cryosections, stained with hematoxylin and eosin (H&E), and then subjected to terminal deoxynucleotidyl transferase dUTP nick-end labeling (TUNEL) for apoptosis analysis.

H&E staining was completed by using a standard protocol for gross histological assessment of cellular density, necrosis, and fibrosis. Images of tumor sections stained with H&E were acquired with a Mirax Scanner (Carl Zeiss, Oberkochen, Germany) by using a 20x objective. The percentage of necrotic area was assessed under a 1000x objective. The degree of apoptosis and tumor necrosis were assessed with TUNEL staining (Roche Diagnostics) according to the manufacturer's instructions. Tumor necrosis rate was measured by ImageJ software (National Institutes of Health). The necrosis rate (%) was calculated by the following formula: necrosis rate (%) = (overall area − survival area)/overall area 100%.

### 2.9. Statistical Analysis

All data were expressed as mean ± standard deviation. An independent sample *t*-test was used to compare the two independent samples, and analysis of variance was used among the three or four groups of the tumor volumes, ablation volumes, and changes of temperature. One-way analysis of variance was performed to analyze ALT and AST levels among the groups. For this test, *P* < 0.05 was considered to indicate statistical significance. All data were analyzed by using SPSS 19.0 (IBM, Armonk, NY, USA).

## 3. Results

We implanted VX2 liver tumors in 26 rabbits and performed conventional grayscale ultrasound and CEUS for the sequential assessment of VX2 tumor growth. The VX2 liver tumor transplantation in 24 (92.3%) rabbits after 14 days of implantation was successful, and these were enrolled in the study.

### 3.1. CEUS Imaging and Volume Change of Unenhanced Perfusion Defects before and after Treatment

**Figure 2:**
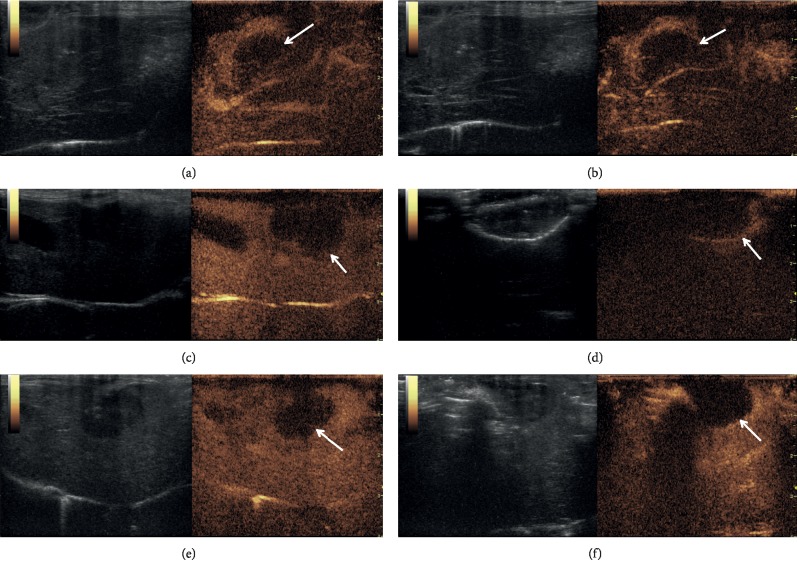
Contrast-enhanced ultrasound images of the tumors from three treatment groups before and after treatment. All liver tumors presented a nonuniform high enhancement model with weak enhancement at the centre (a, c, e) before treatment. After MEUS cavitation, tumor perfusion was decreased and the enlarged unenhanced perfusion defects appeared (b). After MWA ablation, a round-shaped enhancement defect lesion appeared (arrow in (d)). The liver tumor treated by using MEUS + MWA combination showed a much more regular and lower enhancement ablation zone (arrow in (f)).

Posttumor transplantation after 2 weeks revealed that the CEUS images of liver tumors presented a nonuniform high enhancement model with weak enhancement at the centre (Figures 2(a), 2(c), and 2(e)). After treatment, the unenhanced perfusion defects appeared in all the treatment groups, except in the control group (Figures 2(b), 2(d), and 2(f)). The margins of the unenhanced perfusion defects were sharper in the MEUS + MWA group, and their shapes were more regular than those in the other two groups. The maximum length, width, and thickness of the tumors and the unenhanced perfusion defects were determined from the largest slices. The volume was calculated by using the following formula: volume = *π* (length × width × thickness)/6. As no unenhanced perfusion defects were visible in the control group, no measurements were made in this group.  Table 1 shows the tumor volume before treatment and the tumor volume of unenhanced perfusion defects after treatment. The unenhanced perfusion defects of the MEUS group revealed marked differences from those of the MWA and MEUS + MWA groups.

### 3.2. Temperature Changes during Treatment

**Figure 3:**
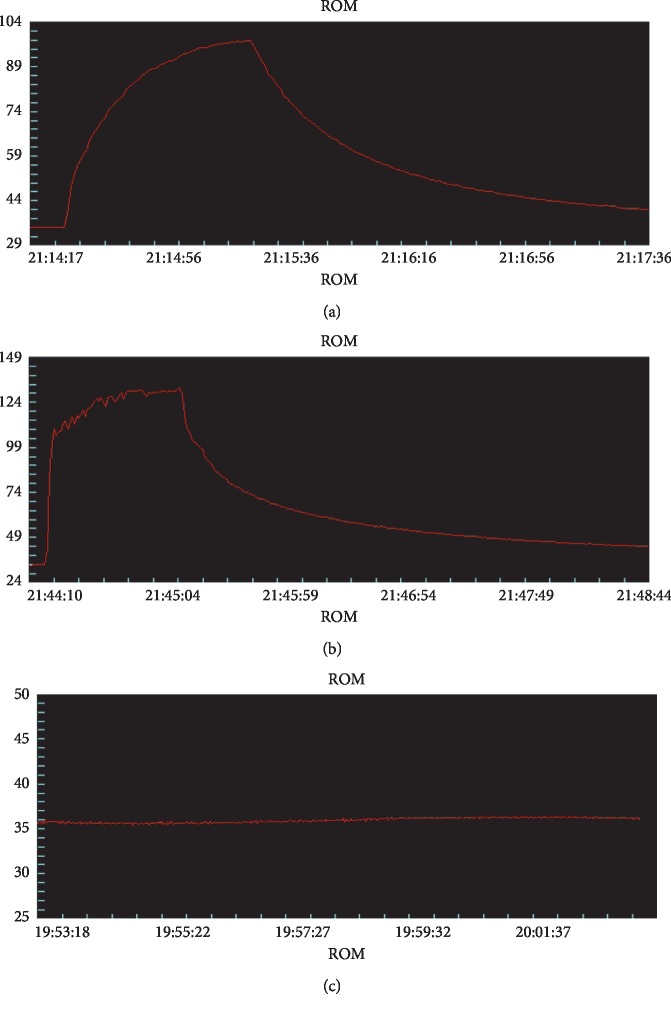
The temperature curves of treatment groups during treatment. The temperature curve during treatment in the MWA group (a) is increased slowly and reached the peak temperature at the end of ablation. The temperature curve during MWA treatment in the MEUA + MWA group (b) is sharply raised at the beginning of ablation, and the peak temperature is obviously higher than that in the MWA group. The temperature curve during treatment in the MEUA + MWA group (b) showed fluctuation during MEUS therapy (c), and there is no significant difference in the temperature fluctuation.

According to the time-temperature curve (Figure 3) the local temperatures of treatment areas were increased slowly during treatments and reached a peak temperature at the end of ablation in the MWA group. The local temperatures of treatment areas were sharply raised at the beginning of ablation and quickly reached a higher peak temperature in the MEUS + MWA group, while local temperatures of treatment areas showed no changes in the MEUS group and in the control group. As shown in Table 2, the peak temperature (PT) in the MEUS + MWA group was obviously higher than that in the MWA group (*P* < 0.01). Compared with the MWA group, the time to reach the peak temperature (TP) in the MEUS + MWA group was shortened (*P* < 0.01).

### 3.3. Changes in Liver Function

After ultrasound cavitation, microwave ablation and combined treatments were performed to examine the liver function. As shown in Table 3, compared with the control group, the values of AST were increased significantly in the three treatment groups, but showed no significant difference between the MWA group and the MEUS + MWA group. The values of ALT showed a significant increase in the MWA group and the MEUS + MWA group, but showed no significant difference between the MWA group and the MEUS + MWA group. There were no statistically significant differences in serum r-GT and TP among all groups.

### 3.4. Histopathological Examination of Liver Tumor and Analysis of Tumor Necrosis Rate

**Figure 4:**
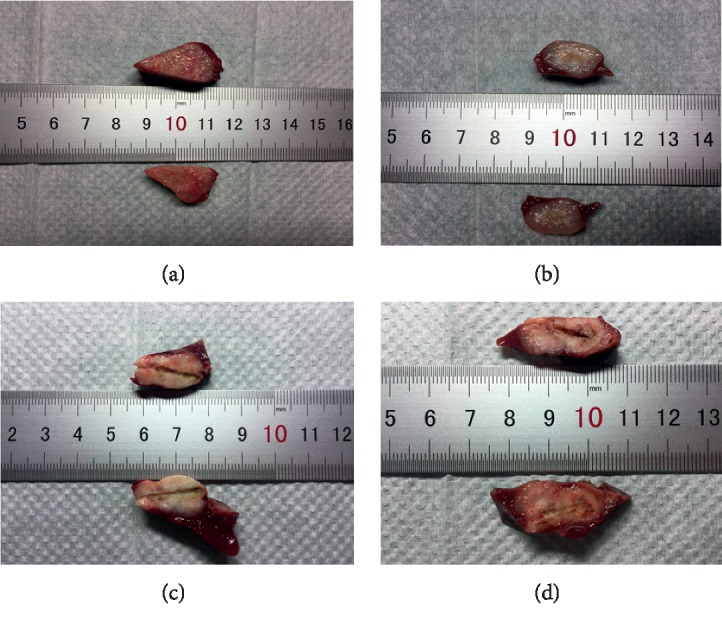
Macroscopic view of liver tumors after treatments. (a) The control group; (b) microbubble-enhanced ultrasound alone; (c) MWA ablation alone; (d) MEUS + MWA ablation. Tumor margins and necrotic margins are difficult to distinguish.

Gross examination of harvested liver and tumor revealed round- or oval-shaped necrotic lesions after treatment by MWA alone or MEUS + MWA combination. Because the tumor margin and necrotic margin are difficult to distinguish (Figure 4), the ablation volumes by gross examination could not be compared.

**Figure 5:**
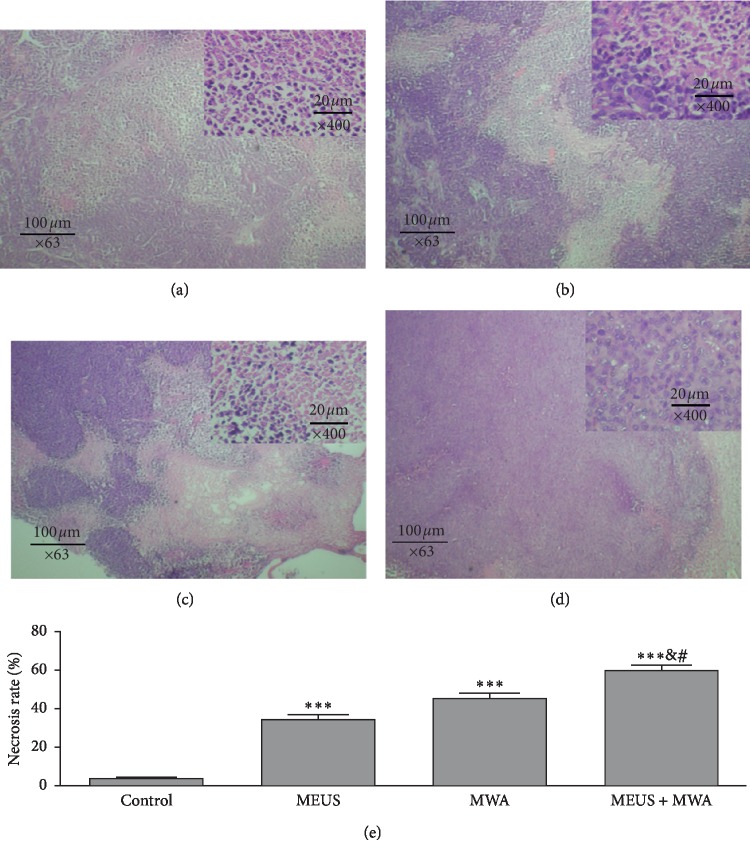
Photographs of hematoxylin and eosin staining. This showed that the boundary between tumor cells and hepatocytes was obvious. Compared with the control group (a), the MEUS group (b), the MWA group (c), and the MEUS + MWA group (d) had cytoplasmic lysis, nuclear condensation and chromatin edge collection, and scattered hemorrhagic spots. The necrosis rate in the MEUS + MWA group was significantly different from the control group (^*∗∗∗*^*P* < 0.05). There was a significant difference between the necrosis rates of the MEUS + MWA group and the other two treatment groups, respectively (^#^*P* < 0.05 and ^&^*P* < 0.05).

Figure 5 illustrates histological changes and necrosis rate in tumor sections in the liver tissues. HE staining showed that the boundary between tumor cells and hepatocytes was obvious. Compared with the normal group, the cavitation group, MWA group, and combined group showed cytoplasmic lysis, nuclear condensation and chromatin edge collection, and scattered hemorrhagic spots.

**Figure 6:**
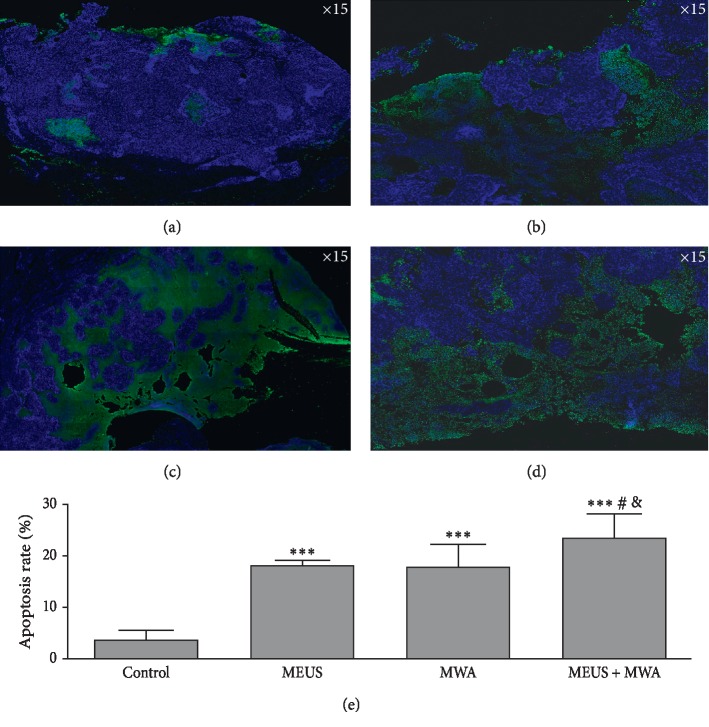
Identification and quantification of TUNEL (terminal deoxynucleotidyl transferase dUTP nick-end labeling) positive VX2 liver tumor cells. (a–d) Representative images from the control group, the MEUS group, the MWA group, and the MEUS + MWA group. TheVX2 liver tumor cells are stained with TUNEL (green) and DAPI (4′,6-diamidino-2-phenylindole, dihydrochloride) (blue), indicating a higher level of apoptosis in the treated tumors relative to the control tumors. The apoptotic rate in the MEUS + MWA group was significantly different from the control group (^*∗∗∗*^*P* < 0.05). There was a significant difference between the apoptotic rates of the MEUS + MWA group and the other two treatment groups, respectively (^#^*P* < 0.05 and ^&^*P* < 0.05).

The percentage of apoptotic cells in tumor sections was measured by calculating the apoptotic index AI) using the number of stained cells with TUNEL and DAPI (examples are provided in Figure 6). Compared with the levels in the other groups (*P* < 0.01) in Figure 6(e), the AI showed the highest in the MEUS + MWA group.

These results indicated that the apoptosis rates of liver tumor cells in the MEUS + MWA group are significantly higher.

## 4. Discussion

This study revealed that the local temperature was rapidly raised to a higher temperature during the MWA ablation on the premise of MEUS, leading to higher apoptosis and necrotic rates of liver tumor cells without damaging the liver function. This in turn determined the feasibility of combining antitumor effects of MEUS with MWA therapy on HCC.

Many studies revealed that MEUS that utilized microbubble-mediated acoustic cavitation can cause endothelial injury, microvascular rupture, tissue edema, hematoma formation, and thrombosis in many tissues and tumors [18, 28]. Our results were consistent with the findings of these studies. In the MEUS alone group, the ultrasound cavitation therapy alone indeed reduced the tumor blood perfusion without accompanying a rise in the temperature. The tumor circulation was stopped (Figure 1(b)) with irregularly unenhanced perfusion defects. The mechanical effects of MEUS produced sonoporation, microvascular rupture, hemorrhage, and microvascular endothelial injury in a variety of tumors [28, 29]. In our study, histopathological examination revealed mild cytoplasmic lysis, nuclear condensation and chromatin edge collection, and scattered hemorrhagic spots. These vascular effects provide an opportunity to overcome the heat sink effect.

It makes sense that MEUS + MWA combination therapy resulted in a rapid rise in the local temperature to a higher peak (Figure 3). This is a very intuitive indication that ultrasound cavitation could enhance the effect of MWA on liver tumors by overcoming the heat sink effect by blocking the blood supply of tumors and promoting the dissolution of tumors. This was also supported by the results of histopathological examination. Previous study [25] showed that the ablation liver volume induced by MEUS + RFA combination therapy was 2.8 times larger than that induced by RFA alone in simple liver tissue, while in the present study, it was difficult to identify the margins of the tumors and the ablation areas, and so the volumes of unenhanced perfusion defects in different groups were compared, which are considered as effective ablation areas. Although there was no statistical difference between the volumes of unenhanced perfusion defects between the MEUS + MWA group and the MWA group, the shape of the ablation area appeared more regular and round and the margin of the ablation area was sharper in the MEUS + MWA group. These phenomena are consistent with the results of histopathological examinations. Pathological staining analysis showed that the necrosis area of VX2 liver tumors in the MWA group was larger than that in the MEUS group, and the necrosis area of the combination therapy group showed the largest, further proving that the combination therapy can rapidly warm up the local temperature to a higher temperature in a short time, and promoting the coagulation as well as the necrosis of tumor tissues. Ultrasound cavitation and MWA can promote the apoptosis and necrosis of tumor tissues. However, no significant differences were observed in the volumes of contrast-enhanced defects before and after MWA in the MWA ablation group and in the MEUS + MWA group. This might be due to the fact that the tumors enrolled in this study and the power and time of microwave treatment were all small. So, it cannot fully reflect the effect of MEUS or MWA.

In this study, the effect on liver function after treatment remains a concern. It was found that MWA and MEUS + MWA can lead to increased ALT and AST levels when compared with the blank control group, which was the manifestation of liver function damage, but there were no significant changes between these two groups by themselves. This suggested that both ultrasound cavitation therapy and MWA therapy and their combination can cause some damages to the liver function, but the damage in the combined group was not obviously higher than those in the alone treatment group. These results demonstrated the safety of combined therapy for treating HCC. Furthermore, previous study [25] found that ALT and AST were peaked at about 24–48 h in the MEUS + RFA and RFA groups, but were almost recovered after 8 d. However, our study was carried out immediately after treatment and cannot reflect the long-term changes of liver function. It is one of the limitations of the present study that we did not continuously observe the liver function after treatment to find out whether the liver function might be restored within a few days.

Regarding the mechanism of the effect of ultrasound cavitation combined with MWA on liver tumors, a series of continuous changes initiated by acoustic cavitation were observed. Firstly, acoustic cavitation enhanced by microbubbles generated mechanical effects, including high-pressure shock waves and microjets [28], leading to transient porosity in the cellular membranes (sonoporation) and damage to the vascular wall. The endothelial cell damage combined with basement membrane exposure is followed by platelet activation, which further promotes the formation of thrombus and increases circulation resistance, and finally reducing or blocking tumor blood perfusion [29]. Finally, the effect of reducing heat sink was achieved. In this study, CEUS showed that blood perfusion in the MEUS + MWA group and the single treatment group was blocked and the blocking effect in the MEUS + MWA group was better when compared to the alone treatment group. Meanwhile, higher PT and shorter time to reach the PT in the MEUS + MWA group showed that ultrasound cavitation could effectively block the blood supply of tumors and surrounding liver tissues, promoting the injury of neovascularization of tumors and significantly reducing the impact of “heat sedimentation effect.” This confirmed that the temperature promotes coagulation and necrosis of tumor tissues.

## 5. Conclusions

Overall, we demonstrated that MEUS can enhance the ablation effect of MWA in rabbit liver tumors without significant damage to the liver function and lead to more complete necrosis and apoptosis of cancer cells. This might be a new approach for the enhancement of MWA that has the potential to achieve more complete ablation of tumors. Further studies to observe the long-term effects of this method such as growth trend, recurrence rate of tumors, and survival rate should be conducted.

## Figures and Tables

**Table 1:** Comparison of tumor volumes (cm^3^) before treatment and volumes (cm^3^) of unenhanced perfusion defects after treatment in each group.

Group	Tumor volume before treatment	Volumes of unenhanced perfusion defects after treatment
Control	1.68 ± 1.04	—
MEUS	1.80 ± 1.03	0.93 ± 0.43^*∗*^
MWA	1.73 ± 1.06	1.78 ± 0.31
MEUS + MWA	1.79 ± 1.05	1.84 ± 0.20

Control = blank control; MEUS = microbubble-enhanced ultrasound; MWA = microwave ablation. ^*∗*^Significantly different from the value of MWA or MEUS + MWA combination treatment.

**Table 2:** Temperature changes during treatments.

Group	TP (s)	PT (°C)
Control	—	34.5 ± 2.0
MEUS	—	34.7 ± 2.0
MWA	21.7 ± 5.0	100.9 ± 5.0
MEUS + MWA	10.3 ± 2.6^*∗*^	134.1 ± 6.0^*∗*^

Control = blank control; MEUS = microbubble-enhanced ultrasound; MWA = microwave ablation; *T* = time to the peak; PT = peak temperature. ^*∗*^Significantly different from the other groups.

**Table 3:** Changes of liver function after treatments.

Group	ALT	AST	r-GT	TP
Control	35.7 ± 6.5^*∗∗*^	43.3 ± 12.1^*∗*^	1.7 ± 0.6^*∗*^	67.0 ± 5.8
MEUS	37.7 ± 13.4	90.5 ± 20.1	5.0 ± 1.9	69.8 ± 3.0
MWA	99.7 ± 25.3	132.2 ± 44.1	5.8 ± 1.5	59.7 ± 9.2
MEUS + MWA	97.0 ± 8.4	135.3 ± 16.5	6.8 ± 3.3	59.6 ± 7.8

^*∗*^Significantly different from the value of other groups. ^*∗∗*^Significantly different from the value of MWA or MEUS + MWA combination treatment.

## Data Availability

The data used to support the findings of this study are available from the corresponding author upon request.
